# Evaluation of the Effectiveness of Crotoxin as an Antiseptic against *Candida* spp. Biofilms

**DOI:** 10.3390/toxins12090532

**Published:** 2020-08-20

**Authors:** Amanda Pissinatti Canelli, Taís Fernanda dos Santos Rodrigues, Vivian Fernandes Furletti de Goes, Guilherme Ferreira Caetano, Maurício Ventura Mazzi

**Affiliations:** 1 Graduate Program of Biomedical Sciences, University Center of Hermínio Ometto Foundation–FHO, Araras, SP, Brazil; apcanelli@gmail.com (A.P.C.); taisrodrigues@alunos.fho.edu.br (T.F.d.S.R.); vivifurletti@fho.edu.br (V.F.F.d.G.); caetanogf@fho.edu.br (G.F.C.); 2 Graduate Program of Orthodontics, University Center of Hermínio Ometto Foundation–FHO, Araras, SP, Brazil

**Keywords:** crotoxin, candida, mouthwash, biofilm

## Abstract

The growing number of oral infections caused by the Candida species are becoming harder to treat as the commonly used antibiotics become less effective. This drawback has led to the search for alternative strategies of treatment, which include the use of antifungal molecules derived from natural products. Herein, crotoxin (CTX), the main toxin of *Crotalus durissus terrificus* venom, was challenged against *Candida tropicalis* (CBS94) and *Candida dubliniensis* (CBS7987) strains by in vitro antimicrobial susceptibility tests. Minimum inhibitory concentration (MIC), minimum fungicidal concentration (MFC), and inhibition of biofilm formation were evaluated after CTX treatment. In addition, CTX-induced cytotoxicity in HaCaT cells was assessed by MTT (3-[4,5-dimethylthiazol-2-yl]-2,5-diphenyltetrazolium bromide) colorimetric assay. Native CTX showed a higher antimicrobial activity (MIC = 47 μg/mL) when compared to CTX-containing mouthwash (MIC = 750 μg/mL) and nystatin (MIC = 375 μg/mL). Candida spp biofilm formation was more sensitive to both CTX and CTX-containing mouthwash (IC100 = 12 μg/mL) when compared to nystatin (IC100 > 47 μg/mL). Moreover, significant membrane permeabilization at concentrations of 1.5 and 47 µg/mL was observed. Native CTX was less cytotoxic to HaCaT cells than CTX-containing mouthwash or nystatin between 24 and 48 h. These preliminary findings highlight the potential use of CTX in the treatment of oral candidiasis caused by resistant strains.

## 1. Introduction

Crotoxin (CTX) is a heterodimeric protein with 16 possible isoforms, consisting of a non-covalent association of a small acidic, non-enzymatic and non-toxic subunit (CA, also named crotapotin) with a basic and weakly toxic phospholipase A2 subunit (CB, also named PLA_2_) [1]. Although extensive research has been carried out on both CTX and its subunits to identify biomedical applications, such as immunomodulatory, antitumor, analgesic, and antimicrobial, the high systemic toxicity of this protein still represents a barrier to its clinical use [2,3].

Considering the therapeutic potential of CTX, the need to develop alternative forms of CTX clinical applications is of high-priority. Chlorhexidine is a classic example of this approach. Considered a broad-spectrum antimicrobial drug with low intestinal absorption, its systemic application is not feasible. However, it is widely used as a topical antiseptic for hand and wound disinfection, and is used in the composition of mouthwashes with a low incidence of toxicity or side effects [4,5].

Dental caries and periodontal diseases are closely related to the formation of supra-gingival and sub-gingival plaques, which are the most significant biofilms in the oral cavity contributing to various orodental manifestations. Both bacterial and yeast species play an important role in this context [6]. Human mucosal surfaces, including the urogenital and gastrointestinal tract, are commonly colonized by Candida species. They can live as commensal microorganisms in healthy individuals, but also contribute to the virulence properties of cariogenic and periodontal bacteria [7,8,9,10].

Besides *Candida albicans*, other Candida species have gained notoriety over the past years. *Candida dubliniensis* is highly prevalent in dental prostheses and has been strongly associated with denture-related stomatitis [11,12,13,14]. Likewise, *Candida tropicalis* is another prevalent non-albicans Candida pathogen that has been shown to express elevated virulence and cause serious disease [15].

Many natural compounds exert anticandidal activity through different mechanisms, providing an important reservoir for the development of antifungal therapies. Alternative therapies are capable of increasing the efficacy and preventing the emergence of drug resistance so that many approaches have been adopted to identify new compounds. Hence, the present work aims to assess the antimicrobial and antibiofilm activities of CTX against both non-albicans Candida species. Moreover, the possibility of incorporating CTX into a mouthwash was also evaluated.

## 2. Results

### 2.1. Isolation and Purification of Crotoxin

**Figure 1:**
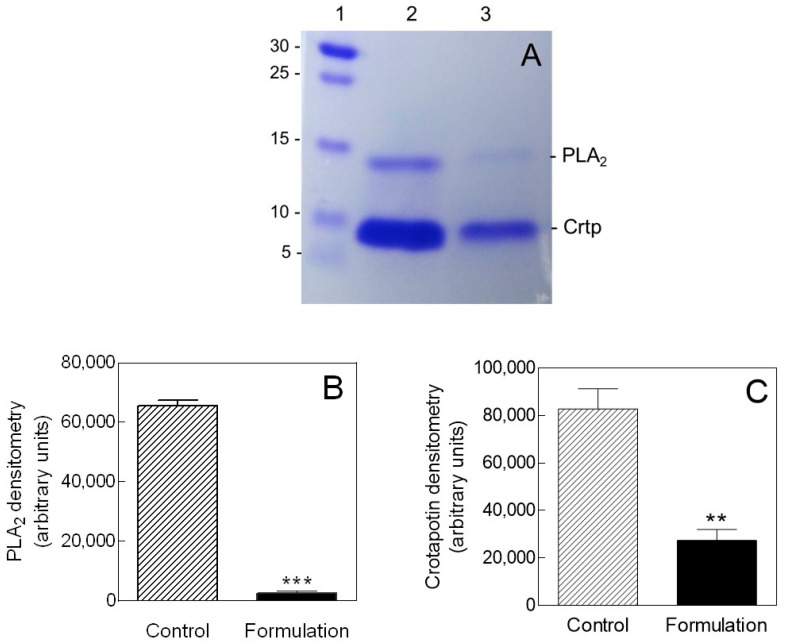
(**A**) Representative electrophoretic profile in SDS-PAGE 13%, under denatured and reduced conditions, of crotoxin purified from *C. durissus terrificus* venom sample. 1: molecular weight markers (kDa); 2: control (10 µg of crotoxin (CTX) in water); 3: formulation (10 µg of CTX in mouthwash). Bar diagrams show arbitrary densitometry units of (**B**) PLA_2_-subunit and (**C**) Crotapotin-subunit as means ± SD (*n =* 3). ** *p* < 0.005 and *** *p* < 0.001 by analysis of variance (ANOVA), followed by Student’s *t*-test. PLA_2_, Phospholipase A_2_; Crtp, crotapotin.

CTX was purified to homogeneity from *C. durissus terrificus* venom. The Coomassie-stained SDS-PAGE gel shows a dimeric protein consisting of two subunits, PLA_2_ and crotapotin, with apparent molecular weights of 14 and 8.9 kDa, respectively (Figure 1A). The same electrophoretic protein profile was obtained for both native CTX (Figure 1A, lane 2) and CTX-containing mouthwash (Figure 1A, lane 3), although a significant loss of protein from the mouthwash was observed (Figure 1B,C).

### 2.2. Antifungal Susceptibility Testing

Broth microdilution assays were performed to investigate the susceptibility of both *C. tropicalis* and *C. dubliniensis* against CTX, CTX-containing mouthwash, basic mouthwash formulation, and nystatin (Table 1). Native CTX showed the highest antimicrobial activity against *C. tropicalis* (minimum inhibitory concentration (MIC) = 47 μg/mL) and *C. dubliniensis* (MIC = 47 μg/mL), exhibiting MIC values 8–16 times lower than those of nystatin (MIC = 375 μg/mL) and CTX-containing mouthwash (MIC = 750 μg/mL), respectively. The highest MIC values were observed for the basic mouthwash formulation (MIC = 1500 µg/mL). Fungal growth in a drug-free complete medium was detected (growth control wells).

Minimum fungicidal concentration (MFC) values for strains subcultured from broth microdilution assays coincided with their corresponding MIC values, except for *C. dubliniensis* treated with CTX-containing mouthwash. In this case, MFC (3000 μg/mL) was four times higher than the corresponding MIC. The MFC/MIC ratios for *C. tropicalis* treated with native CTX and CTX-containing mouthwash were 1:1, suggesting that both displayed fungicidal activity against the strain. The same was observed for *C. dubliniensis* treated with native CTX. On the other hand, CTX-containing mouthwash was most likely to act as fungistatic against *C. dubliniensis*, as its MFC/MIC ratio was >2:1.

### 2.3. Inhibition of Biofilm Formation

**Figure 2:**
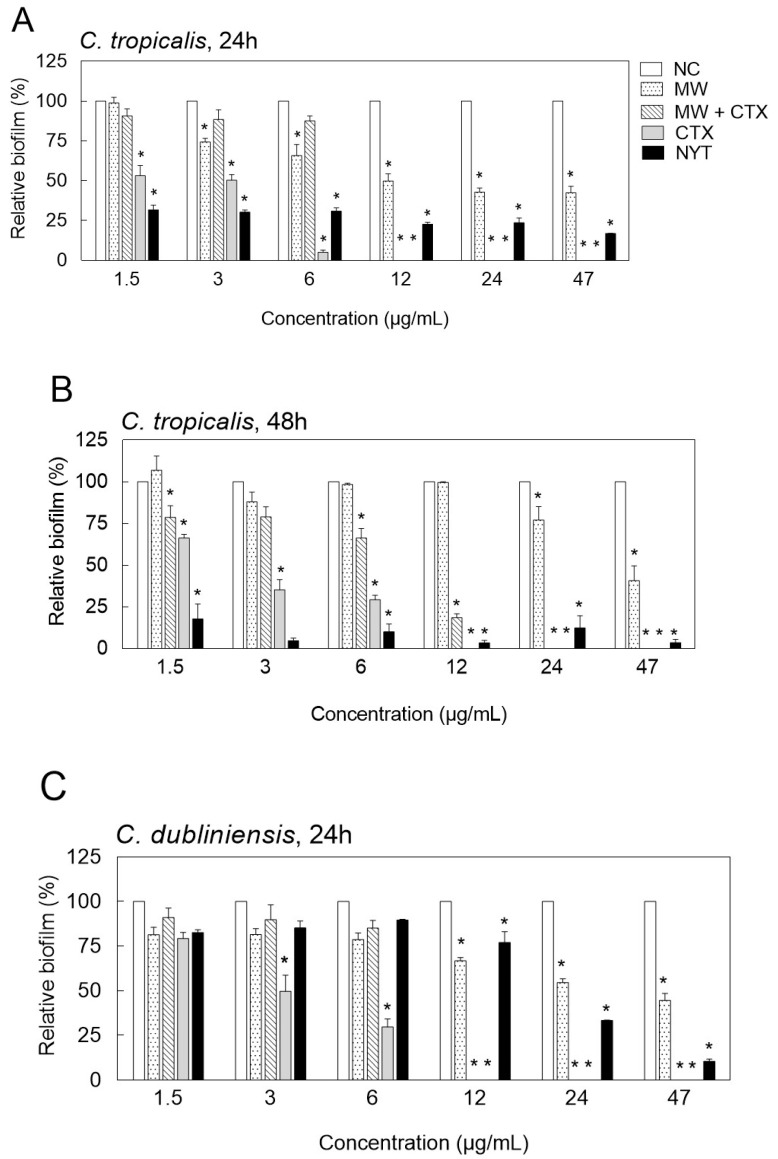

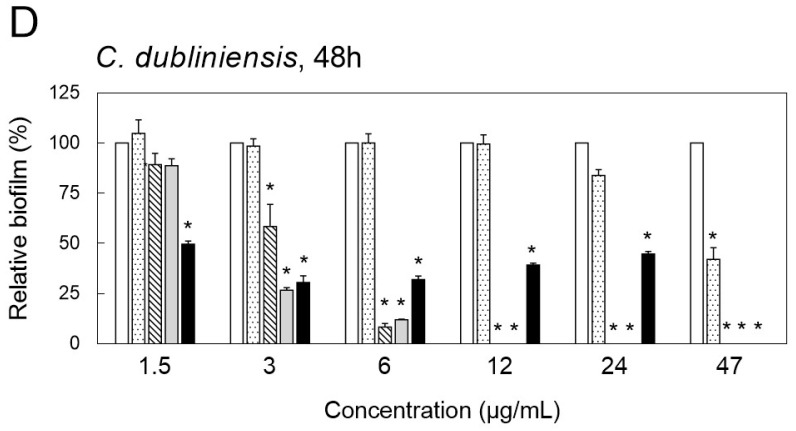
Inhibition of biofilm formation on round bottom polystyrene surface by *Candida tropicalis*, CBS 94 (**A**,**B**) and *Candida dubliniensis*, CBS 7987 (**C**,**D**), after exposure to different compounds and formulations for a period of 24 h (**A**,**C**) and 48 h (**B**,**D**). Data are presented as a percentage of total biomass in treated biofilms and compared to untreated control cell population. Values are means ± SD (*n* = 3). * *p* ≤ 0.0001 vs. untreated group (Tukey’s multiple comparison test). NC, negative control; NYT, nystatin; CTX, native crotoxin; MW + CTX, crotoxin-containing mouthwash; MW, mouthwash.

Figure 2 shows the relative percentage of Candida biofilm mass plotted against increasing concentrations of tested compounds and formulations. Concentrations above 47 µg/mL presented no biofilm formation and therefore were not represented in the plot. Native CTX significantly reduced Candida biofilm formation below 50% at 3 µg/mL, after 48 h of treatment. Both native CTX and CTX-containing mouthwash completely prevented biofilm formation of *C. tropicalis* and *C. dublininensis* at approximately 12 µg/mL, after 24 h of treatment (Figure 2A–D). Similar results were obtained with higher concentrations of nystatin (>47 µg/mL). Nonetheless, lower concentrations of nystatin (<6 µg/mL) were more potent in inhibiting *C. tropicalis* biofilm formation when compared with the same concentration range of native CTX (Figure 2A,B).

**Figure 3:**
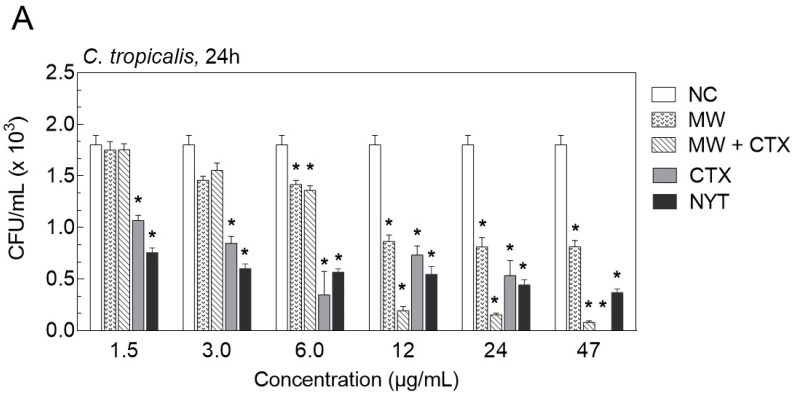

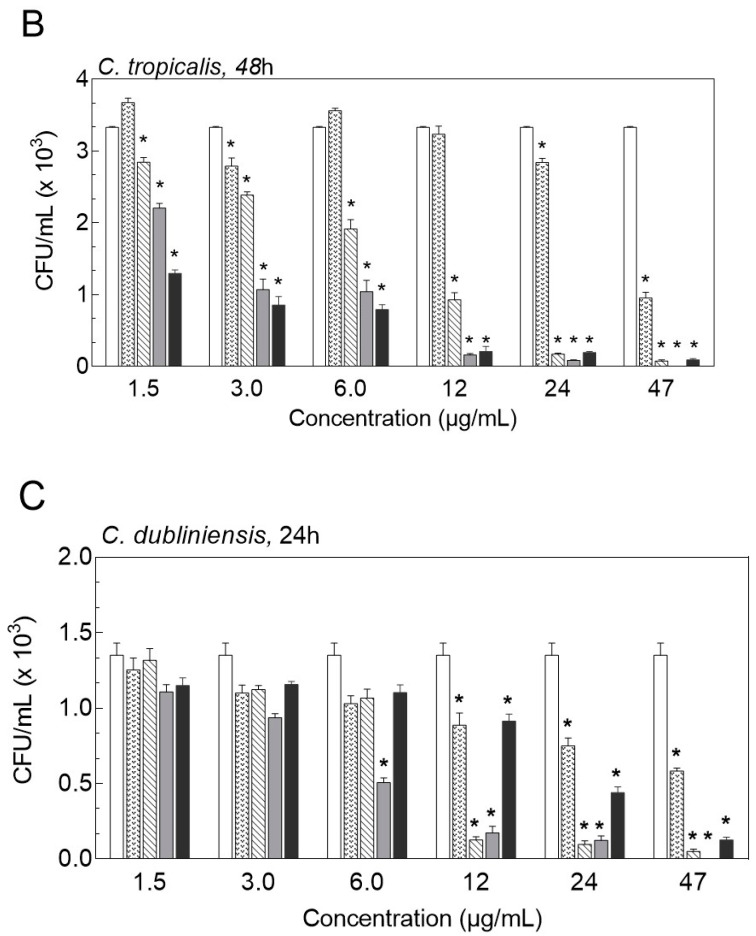

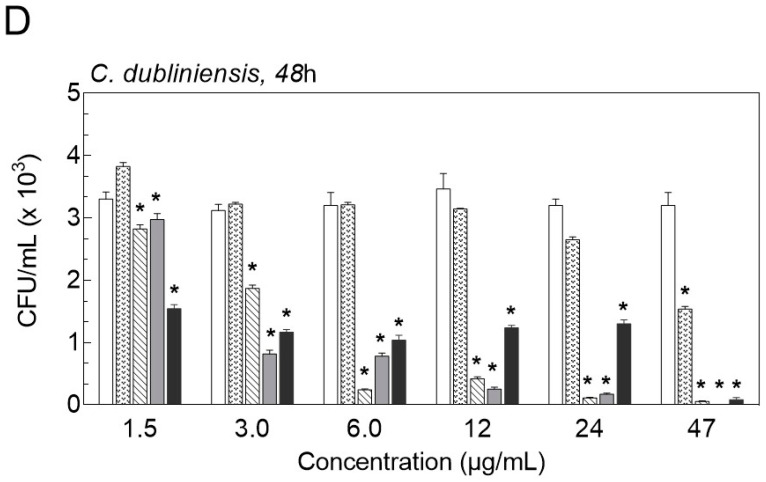
Assessment of the number of colony-forming units (CFU) in biofilms of *Candida tropicalis*, CBS 94 (**A**,**B**), and *Candida dubliniensis*, CBS 7987 (**C**,**D**) challenged with different compounds and formulations for a period of 24 h (**A**,**C**) and 48 h (**B**,**D**). Culture specimens were inoculated onto Sabouraud dextrose agar (SDA), incubated at 36 °C in an aerobic atmosphere for 2 days, and the colonies were counted. Data are presented as means ± SD (*n* = 3) of CFU/mL (×10^3^). * *p* ≤ 0.0001 vs. untreated group (Tukey’s multiple comparison test). NC, negative control; NYT, nystatin; CTX, native crotoxin; MW + CTX, crotoxin-containing mouthwash; MW, mouthwash.

In order to confirm that CTX and CTX-containing mouthwash act as fungistatic or fungicidal, we also determine the viable cells by colony-forming units counting (CFU/mL) after flushing away planktonic forms (Figure 3A–D). We found that the number of CFU/mL of both Candida strains was significantly reduced when 1.5 µg/mL of native CTX was tested, after 24 and 48 h of treatment. The fungicidal effect was observed at the highest concentration of the native CTX (47 μg/mL). In contrast, the same amount of CTX-containing mouthwash presented a fungistatic effect against *C. dubliniensis* and *C. tropicalis*, after 24 and 48 h of treatment, compared to the control. A reduction of 1.7 × 10^3^ CFU/mL of *C. tropicalis* was reached with 47 μg/mL of formulation (Figure 3A,B). Similar results were observed with *C. dubliniensis*, after 24 h (Figure 3C). It was also observed that both Candida strains were more resistant to nystatin compared to native CTX and CTX-containing mouthwash (Figure 3A–D).

### 2.4. Plasma Membrane Integrity

**Figure 4:**
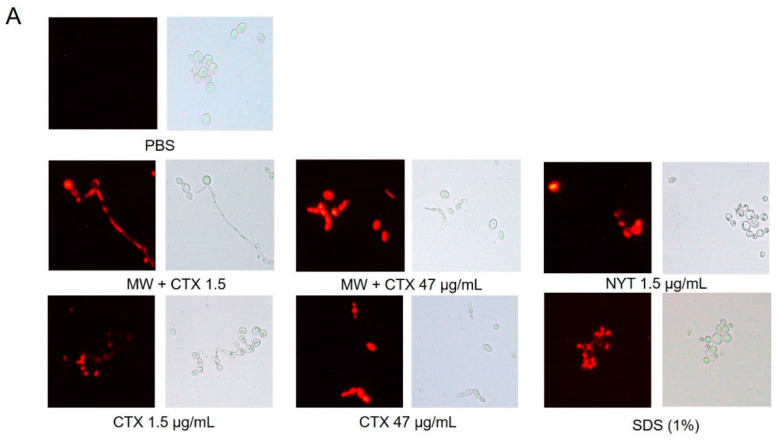

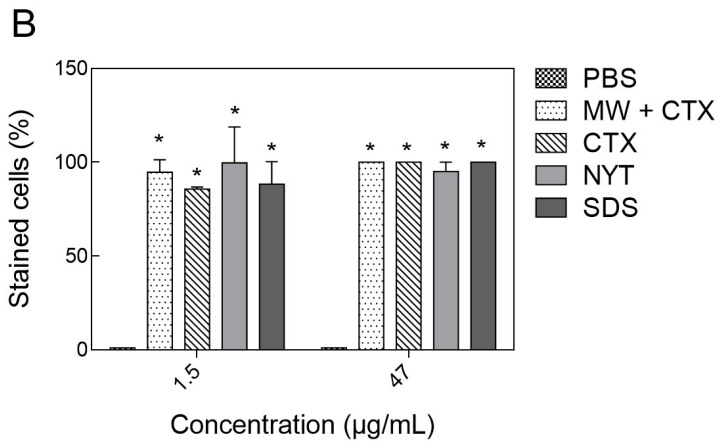
Plasma Membrane Permeability. (**A**) Epifluorescence microscopy images of *C. tropicalis* CBS 94 viability staining with propidium iodide (PI, 6 µM) after treatment with MW + CTX (1.5 and 47 µg/mL), CTX (1.5 and 47 µg/mL) and NYT (1.5 µg/mL). Cells treated with PBS and 1% SDS were used as negative and positive controls of cell membrane permeability, respectively. (**B**) Bar charts represent the percentage of stained cells (dead cells) vs. cells treated with PBS alone. The number of cells were counted in brightfield in 10 random 40× fields, until a total of 100 cells. Each image was independently analyzed for the total area (20 mm^2^) of darkfield/brightfield. Differences were statistically significant (* *p* ≤ 0.001). (magnification, ×40).

**Figure 5:**
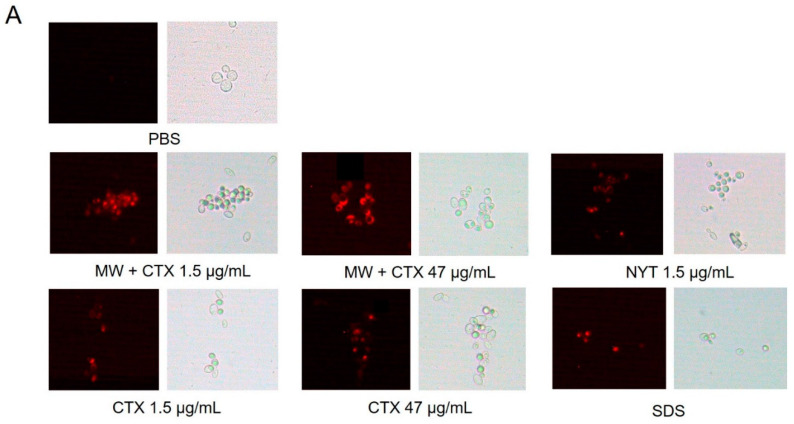

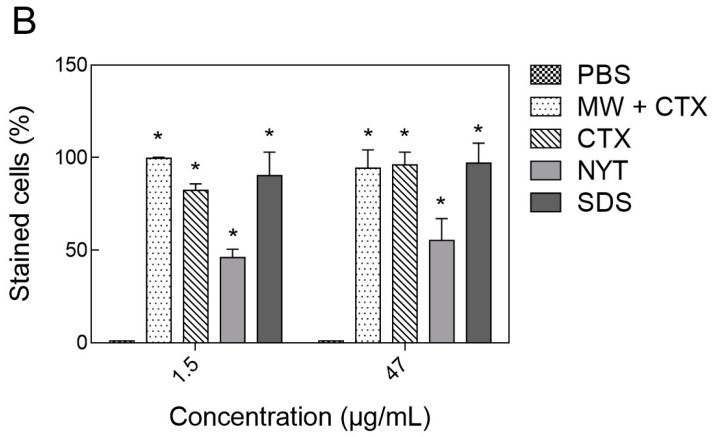
Plasma Membrane Permeability. (**A**) Epifluorescence microscopy images of *C. dubliniensis* CBS 7987 viability staining with propidium iodide (PI, 6 µM) after treatment with MW + CTX (1.5 and 47 µg/mL), CTX (1.5 and 47 µg/mL) and NYT (1.5 µg/mL). Cells treated with PBS and 1% SDS were used as negative and positive controls of cell membrane permeability, respectively. (**B**) Bar charts represent the percentage of stained cells (dead cells) vs. cells treated with PBS alone. The number of cells were counted in brightfield in 10 random 40× fields, until a total of 100 cells. Each image was independently analyzed for the total area (20 mm^2^) of darkfield/brightfield. Differences were statistically significant (* *p* ≤ 0.001). (magnification, ×40).

Plasma membrane permeability assay was performed to assess the in vitro anticandidal mechanism of action of CTX. Native CTX and CTX-containing mouthwash caused membrane permeabilization in both Candida species at concentrations of 1.5 and 47 µg/mL (Figure 4 and Figure 5A). Near 100% of *C. tropicalis* cells challenged with the lowest concentration of CTX were stained with PI (propidium iodide) (Figure 4B). Diversely *C. dubliniensis* cells appeared more resistant to both CTX and nystatin (Figure 5B).

### 2.5. Cell Viability Assay

**Figure 6:**
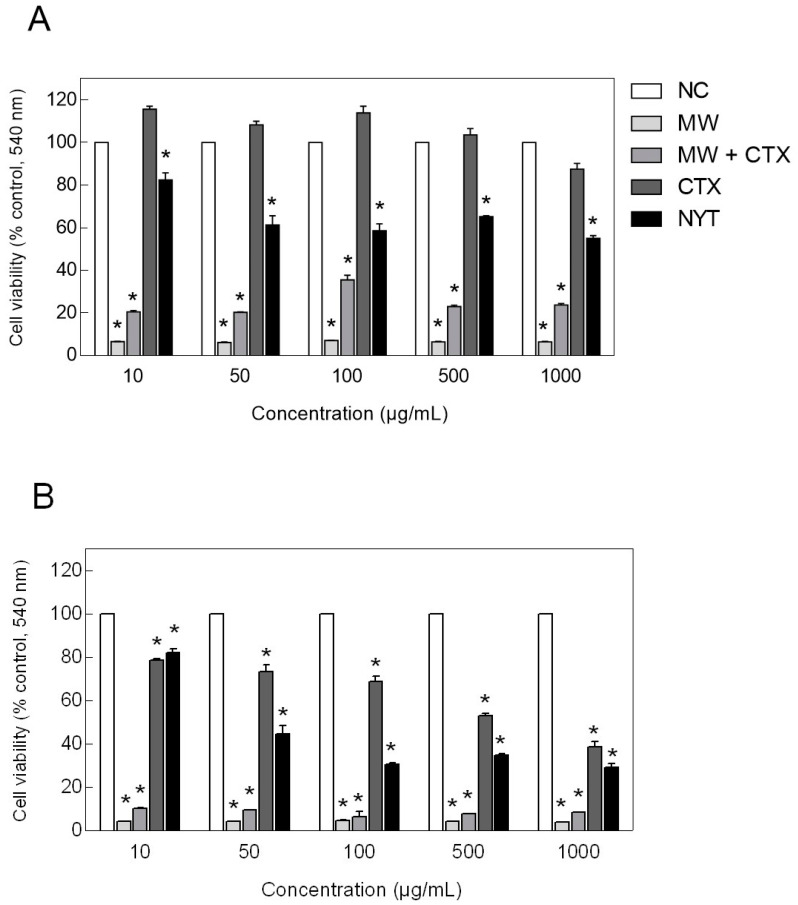
Cytotoxicity assay of HaCaT cell line exposed to CTX and CTX-containing mouthwash. Evaluation of cell viability by MTT assays after exposure for (**A**) 24 h and (**B**) 48 h. Values are expressed as a percentage relative to an undisturbed control cell population (NC). * *p* < 0.0001 vs. untreated group (Tukey’s multiple comparison test). NC, negative control; NYT, nystatin; CTX, native crotoxin; MW + CTX, crotoxin-containing mouthwash; MW, mouthwash.

Cells viability of HaCaT cells was assessed by MTT ((3-[4,5-dimethylthiazol-2-yl]-2,5-diphenyltetrazolium bromide) colorimetric assay. HaCaT cells showed no loss of viability when exposed to native CTX at concentrations ≤500 µg/mL, after a 24-h incubation period (Figure 6A). Instead, cell viability was significantly increased in HaCaT cell cultures exposed to native CTX concentrations ≤100 µg/mL. However, the also revealed that CTX cytotoxicity was significantly increased in a time-dependent manner, as cell viability dropped to approximately 80% to 50% at the same concentration range, after a 48-h exposure period (Figure 6B). HaCaT cells were sensitive to 1000 µg/mL native CTX, showing a decrease in cell viability of approximately 13% and 60%, after 24 and 48 h of treatment, respectively. Even so, native CTX was still less cytotoxic to keratinocytes than the positive control nystatin, at the same concentration range and period of exposure. Additionally, HaCaT cells showed a marked loss of viability when exposed to both CTX-containing mouthwash and basic mouthwash formulation after 24 and 48 h of exposure, suggesting that the observed cytotoxicity is mainly due to formulation components other than CTX.

## 3. Discussion

The search for new synthetic molecules with antifungal potential has increased over the years, mainly due to multiple drug resistance acquired by microorganisms [16]. In seeking to meet this demand, many natural toxins have been widely investigated for their antimicrobial properties, especially against microorganisms of clinical interest [17].

Most studies on snake venom phospholipase A2 (svPLA_2_) published so far report that the bactericidal and/or bacteriostatic properties of these proteins are dose- and time-dependent, with MIC values ranging from 7.5–50 μg/mL. These studies also suggest that bactericidal activity depends on the ability of PLA_2_s to induce changes in membrane permeability [18]. On the other hand, little is known about the fungicidal and fungistatic properties of svPLA_2_ and derived pharmaceuticals.

In this work, crotoxin (CTX), a heterodimeric protein complex formed by the noncovalent association of a PLA2 with a nonenzymatic subunit, was properly purified, incorporated into a mouthwash and challenged against two periodontitis-related strains (*C. tropicalis* and *C. dubliniensis*). Native CTX and CTX-containing mouthwash showed significantly higher antibiofilm effects than nystatin, which served as a positive control treatment. Additionally, native CTX exhibited substantial antimicrobial activity against both strains with equal values of MIC (47 µg/mL). These MIC values were considerably lower when compared with nystatin (MIC = 375 µg/mL) and CTX-containing mouthwash (MIC = 750 µg/mL). However, a significant loss of CTX from the formulation, as evidenced by electrophoretic analysis, may account for the lower antimicrobial activity of CTX-containing mouthwash. This may be possibly caused by interactions of formulation excipients with the protein in solution. Although surfactants have been extensively used in protein-based pharmaceuticals, they may cause the protein molecule to aggregate and precipitate. Moreover, the anionic surfactant sodium lauryl sulfate can interact with the protein’s electrical charges, causing conformational changes that affect its biological activity [19]. Nonetheless, the in vitro antimicrobial and antibiofilm activity of native CTX was superior to that of the conventional nystatin. This suggests a specific mechanism of action that may be related to the membrane damaging activities of the PLA_2_ subunit of CTX [18]. In addition, the differences in the glycerophospholipid compositions found in different Candida species could account for the variations in CTX antimicrobial activity against the two strains investigated herein [20,21,22].

The antimicrobial mechanism of action of svPLA_2_ (particularly anticandidal activity) is still unknown. Cationic antimicrobial proteins, such as svPLA_2_, act by a process of thinning and destabilization of the membrane lipid bilayer, resulting in the permeabilization and expulsion of cellular content. It has been suggested that cationic and hydrophobic regions of svPLA_2_ are responsible for the molecular permeation, due to their high affinity for phospholipid head groups [18]. Furthermore, apart from protein-mediated lysis, it has been proposed that PLA_2_s also inhibit the biosynthesis of macromolecules and promote the expression of autolytic enzymes [18]. Other bacterial membrane structures, such as glucans and ergosterol, have been identified as important targets for antimicrobials as well [23]. Moreover, the amino acid cysteine has been shown to partially inhibit *C. albicans* germination, suggesting that the presence of cysteine residues in both crotapotin and PLA_2_ subunits of CTX would disrupt cell surface hydrophobicity with consequent cell death [18,24]. In this paper, we have demonstrated that *Candida* spp. cells exposed to CTX (at concentrations as low as 1.5 µg/mL) showed altered cell membrane permeability in the same way as for nystatin. Since glycerophospholipid, glucans, and ergosterol are the main components of fungal cell membranes and hence are vital to cellular growth, our results suggest that CTX disrupts the cell membrane integrity and homeostasis to a considerable extent.

As far as the therapeutic use of topical CTX is concerned, the feasibility of its clinical application depends on low selective toxicity. Therefore, cell viability assays were performed to evaluate the in vitro cytotoxic effect of CTX upon a human immortalized keratinocyte cell line (HaCaT). HaCaT cells treated with purified CTX for 24 h displayed higher cell viability than the negative control condition. In a similar study [25], normal keratinocytes (NEHK) and cancer cell lines (SK-LU-1, Hs 578T, and U87-MG) were treated with CTX for 72h at concentrations ranging from 2.3 to 30 µg/mL, and it was demonstrated that NEHK cells were less affected by CTX than cancer cells. In addition, lower CTX concentrations (2.3 to 12.7 µg/mL) were not cytotoxic. In addition, in the present study, it was observed that, unlike native CTX, CTX-containing mouthwash reduced cell viability of HaCaT keratinocytes. In this case, however, cytotoxicity may have been caused by the degenerative effect of sodium lauryl sulfate on cell membranes [26].

In this context, protein carrier systems such as liposomes, cyclodextrins, or PEG conjugates [27,28,29,30] would represent a better alternative to CTX-containing formulations. In a previous report, CTX was successfully encapsulated into sphingomyelin-cholesterol liposomes [27]. It was found that the sphingomyelin/cholesterol liposomal CTX was not toxic when intravenously inoculated in mice at a dose of CTX as high as 91 times its LD_50_, or when subcutaneously inoculated at 42 times its LD_50_. In a similar study, several liposomal formulations containing CTX retained more than 75% of the originally encapsulated protein after 1 week of incubation at physiological temperature, providing a more stable protein formulation. Moreover, an important reduction of CTX toxicity was observed with its encapsulation into liposomes composed of phospholipids that were resistant to PLA_2_ [28]. On the other hand, dextrin conjugation reduced PLA_2_ hemolytic activity but enhanced cytotoxicity in HT29 and in MCF-7 cells, which was partially correlated with epidermal growth factor receptor (EGFR) expression [29].

The proliferative activity induced by CTX would be potentially harmful, if characterized by a marked and persistent increase in cell proliferation. However, the increase in HaCaT cell viability produced by CTX was significant only for a 24 h treatment period, but not after a 48 or 72 h (data not shown) post-exposure period. A CTX-induced keratinocyte proliferation has not been directly reported in the literature, but it is known that the combined use of Gefitinib (trade name Iressa^®^) and CTX (>25 µg/mL) inhibited proliferation of lung cancer cells (A549 strain) without affecting the growth of normal pulmonary fibroblasts [31]. svPLA_2_ catalyzes the hydrolysis of the sn-2 acyl bond of phospholipids, releasing arachidonic acid (AA) and lysophosphatidic acid. Upon downstream enzymatic modification, AA is modified into active compounds, which can exert a wide range of physiological and pathological effects, including inflammatory response, pain perception, and cell proliferation [32,33]. Nonetheless, several studies have also demonstrated that CTX can modulate immune responses and exert anti-inflammatory and immunosuppressive effects [34]. Further, CTX was shown to modulate calcium signaling [2,34,35], which may account for the increase in HaCaT cell viability induced by low concentrations of CTX. The elucidation of such an effect would require more detailed studies concerned with the complex intracellular signaling pathways.

## 4. Conclusions

This is a preliminary study for the assessment of the antimicrobial and antibiofilm activities of native CTX and a CTX-containing mouthwash upon two Candida species of clinical interest. Our data indicate that CTX is fungicidal rather than fungistatic, which may help solve the issue of innate or acquired drug resistance due to the prophylactic use of fungistatic drugs. Some of the limitations associated with this study are those related to the chemical composition of the mouthwash affecting protein stability, and the small number of Candida strains evaluated. The use of liposomes or cyclodextrins complexes as protein carriers might increase the stability of CTX and provide its controlled release. Thus, future studies should include the development of more elaborate therapeutic mouthwashes, in vivo assays using the experimental model of oral candidiasis in rats, and a comprehensive analysis of the mode of action and topical toxicity of CTX.

## 5. Material and Methods

This study was approved by the Institutional Research Ethics Committee under consubstantiated opinion nº 008/2018 as of 13/03/2018.

### 5.1. Purification of Crotoxin

The yellowish venom of *Crotalus durissus terrificus* (South American rattlesnake) was purchased from Koemitã (Mococa, São Paulo, Brazil). CTX was isolated and purified from the *Crotalus durissus terrificus* venom through two steps of DEAE-Sepharose and Heparin Sepharose FF chromatography, and protein identification was performed in a Waters^®^ Q-Tof Premier™ Mass Spectrometer, nanoACQUITY UPLC™ system, using a silica-based C18 Atlantis™ column (Waters Corporation, Milford, MA, USA), as extensively described previously [36]. The purity of CTX was determined by 13% (*w*/*v*) SDS-PAGE in a Tris-glycine buffer of pH 8.3, for 120 min at 30 mA/100 V. The molecular mass was measured using the Carestream Molecular Imaging Software (Carestream Health Inc., Rochester, NY, USA) and at 5–40 kDa molecular weight calibration standards PageRuler Low Range Unstained Protein Ladder (Thermo Fisher Scientific, Waltham, MA, USA).

### 5.2. Preparation of a Crotoxin-Containing Mouthwash

The test mouthwash containing CTX was prepared by the investigator at the Department of Pharmaceutical Sciences, Hermínio Ometo Foundation, Araras (SP), Brazil. The formulation was composed of a combination of the purified and freeze-dried CTX (1.2% *w*/*v*), sorbitol (20% *w*/*v*), and sodium lauryl sulfate (1% *w*/*v*) in enough purified water to make 100 mL.

### 5.3. Strains and Culture Conditions

*Candida dubliniensis* (CBS 7987) and *Candida tropicalis* (CBS 94) were used throughout this study, and were supplied by reference strains obtained from the Netherlands Collection—CBS. Strains were stored and maintained on Sabouraud dextrose agar (SDA), before propagation in Sabouraud dextrose broth (SDB) for 16 to 18 h, at 36 °C, with gentle agitation. All culture media were manufactured by KASVI (São José dos Pinhais, Paraná, Brazil).

Human immortalized non-tumorigenic keratinocyte cell line HaCaT, (ethnicity, Caucasian; age, 62 years; gender, male; and tissue, skin) were supplied by Ribeirão Preto Medical School (Internal Medicine Department, University of São Paulo). Dulbecco’s modified eagle medium, containing 4.5 g/L d-glucose, l-glutamine, and 110 mg/L sodium pyruvate (DMEM high glucose; Invitrogen) supplemented with 2 mM l-glutamine, 10% heat-inactivated fetal bovine serum (FBS) and penicillin-streptomycin (100 U/mL, 0.1 mg/mL) was used as a culture medium (Biotech Inc., Oklahoma City, OK, USA). Cells were incubated at 37 °C for 24 h with 5% CO_2_ in humidified air. The medium was changed every 2 days and cells were tested when 70% confluence.

### 5.4. Antifungal Susceptibility Testing

Fungal susceptibility to CTX and CTX-containing mouthwash was determined according to the CLSI M27-A2 broth microdilution reference method [37], with minor modifications [38], and performed on sterile 96-well round-bottom microplates. CTX and CTX-containing mouthwash were diluted with SDB to achieve 2X concentrations of the protein (6000 to 3 µg/mL), and a volume of 100 μL was dispensed into the wells. Yeast suspensions were prepared in 0.85% sterile saline from 24-h SDA plates and adjusted to the turbidity of a 0.5 McFarland standard (0.08–0.10 at 530 nm), which is equivalent to 1 × 10^6^ to 5 × 10^6^ CFU/mL. These suspensions were further diluted with culture medium to obtain the concentrated 2X inoculum used in the test (1 × 10^4^ to 5 × 10^4^ CFU/mL). Each well of the microdilution plate was inoculated with 100 µL of the corresponding concentrated 2X suspension to reach the desired final concentrations of inoculum and CTX (3000 a 1.5 µg/mL). Serial dilutions of the basic mouthwash formulation were also tested. Nystatin (3000 a 1.5 µg/mL, Nova Química Farmacêutica Ltd.a, Barueri, São Paulo, Brazil) served as a positive control. Growth control wells contained 100 µL of drug-free sterile medium inoculated with 100 µL of the 2X concentrated inoculum suspensions. The inoculated plates were incubated in an aerobic atmosphere at 36 °C for 24 and 48 h. Results were interpreted as per CLSI guidelines. Minimum inhibitory concentration (MIC) was determined as the lowest concentration of antimicrobial agent that prevented visible growth of the microorganism after incubation. Afterward, minimum fungicidal concentration (MFC) was determined by subculturing 10 µL from each well showing no apparent growth on SDA plates, at 36 °C for 24 and 48 h. MFC was considered as the lowest concentration of antimicrobial agents yielding negative subcultures or only one colony on SDA after incubation. The MFC/MIC ratios were determined for each strain and treatment, to specify the nature of the antimicrobial effect, as described before [39]. All experiments were performed in triplicate for three independent experiments.

### 5.5. Biofilm Growth on Polystyrene and Adherence Inhibition

Biofilm growth of *Candida* spp. on the sterile 96-well, round-bottom polystyrene microplates was performed similarly as described for the antifungal susceptibility testing, with modifications described by Peters et al. [40]. Briefly, CTX and CTX-containing mouthwash were diluted with SDB to achieve 2X concentrations of CTX (6000 to 3 µg/mL), and a volume of 100 μL was dispensed into the wells. Then, 100 µL of adjusted *Candida* sp. suspension (1 × 10^6^ CFU/mL) was added to each well of the microdilution plate to reach the desired final concentrations of inoculum and CTX. Plates were incubated for 24 and 48 h at 36 °C, with no agitation to induce biofilm formation. Controls included serial dilutions of the basic mouthwash formulation and nystatin. Growth control wells contained 100 µL of drug-free sterile medium inoculated with 100 µL of a 1 × 10^6^ CFU/mL inoculum. In order to quantify biomass during yeast growth and to evaluate biofilm adherence inhibition, biofilms were processed for crystal violet staining. The liquid content of microplates was discarded and wells were washed gently in PBS to remove non-adherent cells. Microplates were inverted to drain on filter paper for 20 min. Then, each well was stained with 1% crystal violet (150 µL) for 30 min and repeatedly washed in distilled H_2_O. Microplates were once again inverted to drain on filter paper for 20 min. Bound crystal violet was resolubilized in 95% ethanol, and the absorbance was read at 540 nm on a BioTek ELx800^®^ microplate reader (BioTek^®^ Instruments, Inc., Winooski, VT, USA). Results were expressed as a percentage, relative to the growth control. The inhibition of biofilm formation was calculated relative to the amount of biofilm that was grown in the absence of any active compound (defined as 100% biofilm) and the media sterility control (defined as 0% biofilm). Results from at least three separate biological replicates were averaged. All samples were also assessed for colony-forming unit counts. In this case, the same experiment was performed. After incubation with active compounds, planktonic cells were aspirated and the wells were washed to remove non-adherent cells. The adhered cells from each well were resuspended in sterile PBS (500 µL) and seeded (100 µL) in SDA plates, followed by incubation at 36°C for 24h, under an aerobic atmosphere. The colonies were counted and the results were expressed as colony-forming units (CFU/mL). Results from at least three separate biological replicates were averaged.

### 5.6. Plasma Membrane Permeability

Plasma membrane integrity was assessed as described by Suchodolski et al. [41]. Briefly, *C. albicans* and *C. dubliniensis* cells were harvested by centrifugation (10 min, 956× *g*) in the stationary phase and washed twice with PBS, followed by 2 h of incubation in PBS (1 × 10^6^ CFU/mL) with different concentrations of CTX and CTX-containing mouthwash (1.5 and 47 µg/mL). Cells were then washed twice with PBS, resuspended in 200 µL PI (6.0 µM), and incubated for 5 min, at room temperature. After incubation, treated cells were washed twice again with PBS. The pellets were resuspended in 0.25 mL of PBS and 4 µL of samples were visualized with a LEICA^®^ DM-2000 optical microscope coupled with a LEICA^®^ DFC-280 camera and LAS^®^ version 3.3.0 software (Leica Microsystem, Wetzlar, Germany). Images were captured at 40× magnification, under standardized microscopy parameters. The Plugin “Cell Counter” from ImageJ software was used for counting of cells on 3 images from 3 different experiments. The number of cells were counted in brightfield in 10 random 40× fields, until a total of 100 cells. Each image was independently analyzed for the total area (20 mm^2^) of darkfield/brightfield and results expressed by the percentage of stained cells. The percentage of cell death was assessed by measuring the area ratio of the fluorescent regions to the dark regions (a mask of the bright field was subtracted from the fluorescent image to isolate the objects under analysis). Cell death control (positive control) consisted of cells treated with 1% SDS, while cell viability control (negative control) consisted of cells mock-treated with PBS alone.

### 5.7. Cell Viability Assay

HaCaT cell viability was used as an indicator of cytotoxicity. It was determined by the MTT colorimetric assay as described elsewhere [42], in the presence of CTX or CTX-containing mouthwash. HaCaT cells were seeded into 96-well plates at a density of 3 × 10^4^ cells/well with DMEM complete medium (200 μL of medium/well). After 24 h, the culture medium was replaced with fresh complete medium containing serial dilutions of CTX aqueous solution or CTX-containing mouthwash to achieve CTX final concentrations of 1000, 500, 100, 50, and 10 µg/mL. Cells were incubated for 24 and 48 h, after which incubation medium was removed and replaced by 200 μL of fresh medium. Then, MTT reagent (3-[4,5-dimethylthiazol-2-yl]-2,5-diphenyltetrazolium bromide, Sigma-Aldrich, St. Louis, MO, USA) was dissolved in PBS, 20 μL of stock MTT solution was added to culture wells at 0.5 mg/mL final concentration, and plates were incubated at 37 °C for 3 h. After incubation, the supernatant was carefully removed; the purple-blue formazan salt crystals formed were dissolved in 200 µL DMSO (Sigma-Aldrich, St. Louis, MO, USA), and measured spectrophotometrically at 540 nm using a Varioskan LUX™ Multimode microplate reader (Thermo Fisher Scientific, Waltham, MA, USA). Each experiment was performed three times in triplicate. Cell viability was expressed as a percentage relative to an undisturbed control cell population (HaCaT cells cultured in DMEM complete medium), which served to mark the 100% cell viability (negative control). As a positive control for toxicity, HaCaT cells were cultured in DMEM complete medium supplemented with DMSO (1:1). The IC50 values were calculated by GraphPad Prism 6.0 software (GraphPad Software Inc., San Diego, CA, USA).

### 5.8. Statistical Analysis

Data are expressed as mean ± SD of at least three independent experiments performed in triplicate. Data were analyzed by unpaired two-way analysis of variance (ANOVA), followed by Tukey’s multiple comparison test. Statistical analysis was done using GraphPad Prism 6.0 software (GraphPad Software Inc., San Diego, CA, USA). *p* < 0.05 was considered statistically significant.

## Figures and Tables

**Table 1:** Minimum inhibitory and minimum fungicidal concentrations of CTX and CTX-containing mouthwash against two Candida species.

Yeast Strain	NYT		CTX		MW + CTX		MW		*p* Value
	MIC	MFC	MIC	MFC	MIC	MFC	MIC	MFC	
*C. tropicalis*	375	375	47	47	750	750	1500	1500	<0.05
*C. dubliniensis*	375	375	47	47	750	3000	1500	1500	<0.05

MIC (minimal inhibitory concentration, μg/mL); MFC (minimal fungicidal concentration, μg/mL); NYT, nystatin; CTX, native crotoxin; MW + CTX, crotoxin-containing mouthwash; MW, mouthwash.

## References

[1] Faure G., Saul F. (2012). Crystallographic characterization of functional sites of crotoxin and ammodytoxin, potent β-neurotoxins from Viperidae venom. Toxicon.

[2] Sampaio S.C., Hyslop S., Fontes M.R., Prado-Franceschi J., Zambelli V.O., Magro A.J., Brigatte P., Gutierrez V.P., Cury Y. (2010). Crotoxin: Novel activities for a classic beta-neurotoxin. Toxicon.

[3] Teixeira N.B., Sant’Anna M.B., Giardini A.C., Araujo L.P., Fonseca L.A., Basso A.S., Cury Y., Picolo G. (2020). Crotoxin down-modulates pro-inflammatory cells and alleviates pain on the MOG35-55-induced experimental autoimmune encephalomyelitis, an animal model of multiple sclerosis. Brain Behav. Immun..

[4] Lim K.S., Kam P.C. (2008). Chlorhexidine—Pharmacology and clinical applications. Anaesth. Intensive Care.

[5] Fiorillo L. (2019). Chlorhexidine gel use in the oral district: A systematic review. Gels.

[6] Larsen T., Fiehn N.E. (2017). Dental biofilm infections—An update. Apmis.

[7] Bizerra F.C., Nakamura C.V., de Poersch C., Estivalet Svidzinski T.I., Borsato Quesada R.M., Goldenberg S., Krieger M.A., Yamada-Ogatta S.F. (2008). Characteristics of biofilm formation by *Candida tropicalis* and antifungal resistance. FEMS Yeast Res..

[8] Peters B.M., Jabra-Rizk M.A., O’May G.A., Costerton J.W., Shirtliff M.E. (2012). Polymicrobial interactions: Impact on pathogenesis and human disease. Clin. Microbiol. Rev..

[9] Allison D.L., Willems H.M.E., Jayatilake J.A.M.S., Bruno V.M., Peters B.M., Shirtliff M.E. (2016). *Candida*-Bacteria Interactions: Their Impact on Human Disease. Microbiol. Spectr..

[10] Pereira D., Seneviratne C.J., Koga-Ito C.Y., Samaranayake L.P. (2018). Is the oral fungal pathogen *Candida albicans* a cariogen?. Oral Dis..

[11] Gutiérrez J., Morales P., González M.A., Quindós G. (2002). *Candida dubliniensis*, a new fungal pathogen. J. Basic Microbiol..

[12] Gasparoto T.H., Dionísio T.J., de Oliveira C.E., Porto V.C., Gelani V., Santos C.F., Campanelli A.P., Lara V.S. (2009). Isolation of *Candida dubliniensis* from denture wearers. J. Med. Microbiol..

[13] Meurman J.H., Pärnänen P., Seneviratne C.J., Samaranayake L.P., Saarinen A.M., Kari K. (2010). Prevalence and antifungal drug sensitivity of non-albicans Candida in oral rinse samples of self-caring elderly. Gerodontology.

[14] Zomorodian K., Haghighi N.N., Rajaee N., Pakshir K., Tarazooie B., Vojdani M., Sedaghat F., Vosoghi M. (2011). Assessment of Candida species colonization and denture-related stomatitis in complete denture wearers. Med. Mycol..

[15] Kothavade R.J., Kura M.M., Valand A.G., Panthaki M.H. (2010). *Candida tropicalis*: Its prevalence, pathogenicity and increasing resistance to fluconazole. J. Med. Microbiol..

[16] Ortiz A., Sansinenea E. (2019). The Chemistry of Drugs to Treat *Candida albicans*. Curr. Top. Med. Chem..

[17] Primon-Barros M., Macedo A.J. (2017). Animal Venom Peptides: Potential for new antimicrobial agents. Curr. Top. Med. Chem..

[18] Almeida J.R., Palacios A.L.V., Patiño R.S.P., Mendes B., Teixeira C.A.S., Gomes P., da Silva S.L. (2019). Harnessing snake venom phospholipases A_2_ to novel approaches for overcoming antibiotic resistance. Drug Dev. Res..

[19] Naidu K.T., Prabhu N.P. (2011). Protein-surfactant interaction: Sodium dodecyl sulfate-induced unfolding of ribonuclease A. J. Phys. Chem. B.

[20] Singh A., Prasad T., Kapoor K., Mandal A., Roth M., Welti R., Prasad R. (2010). Phospholipidome of Candida: Each species of Candida has distinctive phospholipid molecular species. OMICS J. Integr. Biol..

[21] Raut J., Rathod V., Karuppayil S.M. (2010). Cell surface hydrophobicity and adhesion: A study on fifty clinical isolates of *Candida albicans*. Nihon Ishinkin Gakkai Zasshi.

[22] Yadav U., Khan M.A. (2018). Targeting the GPI biosynthetic pathway. Pathog. Glob. Health.

[23] Magnani M., Castro-Gómez R.J.H. (2008). Beta-glucana from Saccharomyces cerevisiae: Constitution, bioactivity and obtaining. Semin. Ciênc. Agrár..

[24] Faure G., Guillaume J.L., Camoin L., Saliou B., Bon C. (1991). Multiplicity of acidic subunit isoforms of crotoxin, the phospholipase A_2_ neurotoxin from *Crotalus durissus terrificus* venom, results from posttranslational modifications. Biochemistry.

[25] Rudd C.J., Viskatis L.J., Vidal J.C., Etcheverry M.A. (1994). In vitro comparison of cytotoxic effects of crotoxin against three human tumors and a normal human epidermal keratinocyte cell line. Investig. New Drugs.

[26] Cvikl B., Lussi A., Gruber R. (2015). The in vitro impact of toothpaste extracts on cell viability. Eur. J. Oral Sci..

[27] Freitas T.V., Frézard F. (1997). Encapsulation of native crotoxin in liposomes: A safe approach for the production of antivenom and vaccination against *Crotalus durissus terrificus* venom. Toxicon.

[28] Magalhães T., Proietti Viotti A., Teperino Gomes R., Viana de Freitas T. (2001). Effect of membrane composition and of co-encapsulation of immunostimulants in a liposome-entrapped *crotoxin*. Biotechnol. Appl. Biochem..

[29] Ferguson E.L., Richardson S.C.W., Duncan R. (2010). Studies on the mechanism of action of dextrin−phospholipase A_2_ and its suitability for use in combination therapy. Mol. Pharm..

[30] Bianco I.D., Daniele J.J., Delgado C., Fisher D., Francis G.E., Fidelio G.D. (2002). Coupling reaction and properties of poly(ethylene glycol)-linked phospholipases A_2_. Biosci. Biotechnol. Biochem..

[31] Ye B., Xie Y., Qin Z.H., Wu J.C., Han R., He J.K. (2011). Anti-tumor activity of CrTX in human lung adenocarcinoma cell line A549. Acta Pharmacol. Sin..

[32] Farooqui A.A., Horrocks L.A. (2006). Phospholipase A_2_-generated lipid mediators in the brain: The good, the bad, and the ugly. Neuroscientist.

[33] Murakami M., Taketomi Y. (2015). Secreted phospholipase A_2_ and mast cells. Allergol. Int..

[34] Sartim M.A., Menaldo D.L., Sampaio S.V. (2018). Immunotherapeutic potential of Crotoxin: Anti-inflammatory and immunosuppressive properties. J. Venom. Anim. Toxins Trop. Dis..

[35] Colombo I., Sangiovanni E., Maggio R., Mattozzi C., Zava S., Corbett Y., Fumagalli M., Carlino C., Corsetto P.A., Scaccabarozzi D. (2017). HaCaT cells as a reliable in vitro differentiation model to dissect the inflammatory/repair response of human keratinocytes. Mediat. Inflamm..

[36] Muller S.P., Silva V.A.O., Silvestrini A.V.P., de Macedo L.H., Caetano G.F., Reis R.M., Mazzi M.V. (2018). Crotoxin from *Crotalus durissus terrificus* venom: In vitro cytotoxic activity of a heterodimeric phospholipase A_2_ on human cancer-derived cell lines. Toxicon.

[37] CLSI (2017). Reference Method for Broth Dilution Antifungal Susceptibility Testing of Yeasts.

[38] Marak M.B., Dhanashree B. (2018). Antifungal Susceptibility and Biofilm Production of Candida spp. Isolated from Clinical Samples. Int. J. Microbiol..

[39] Hafidh R.R., Abdulamir A.S., Vern L.S., Abu Bakar F., Abas F., Jahanshiri F., Sekawi Z. (2011). Inhibition of growth of highly resistant bacterial and fungal pathogens by a natural product. Open Microbiol. J..

[40] Peters B.M., Ward R.M., Rane H.S., Lee S.A., Noverr M.C. (2013). Efficacy of ethanol against *Candida albicans* and *Staphylococcus aureus* polymicrobial biofilms. Antimicrob. Agents Chemother..

[41] Suchodolski J., Feder-Kubis J., Krasowska A. (2017). Antifungal activity of ionic liquids based on (-)-menthol: A mechanism study. Microbiol. Res..

[42] Denizot F., Lang R. (1986). Rapid colorimetric assay for cell growth and survival: Modifications to the tetrazolium dye procedure giving improved sensitivity and reliability. J. Immunol. Methods.

